# Supplementary material for: Multi‐dataset identification of innovative feature genes and molecular mechanisms in keratoconus
Source: J Cell Mol Med. 2024 Sep 19;28(18):e70079. doi: 10.1111/jcmm.70079 (PMC11412914; doi:10.1111/jcmm.70079)
Supplement: Supplementary file 2 — Material S2. [file JCMM-28-e70079-s002.zip › 7.GSEA analysis/5.RNF217-AS1/KEGG_GLUTATHIONE_METABOLISM.html]

Details for gene set KEGG\_GLUTATHIONE\_METABOLISM[GSEA]

|  || Dataset | RNF217-AS1.group.cls#h\_versus\_l |
| Phenotype | group.cls#h\_versus\_l |
| Upregulated in class | l |
| GeneSet | KEGG\_GLUTATHIONE\_METABOLISM |
| Enrichment Score (ES) | -0.41681212 |
| Normalized Enrichment Score (NES) | -1.3861705 |
| Nominal p-value | 0.12810707 |
| FDR q-value | 0.37023762 |
| FWER p-Value | 0.884 |
Table: GSEA Results Summary

  

Fig 1: Enrichment plot: KEGG\_GLUTATHIONE\_METABOLISM      
 Profile of the Running ES Score & Positions of GeneSet Members on the Rank Ordered List

  

| SYMBOL | TITLE | RANK IN GENE LIST | RANK METRIC SCORE | RUNNING ES | CORE ENRICHMENT || 1 | GPX6 | na | 5046 | 0.215 | -0.1036 | No |
| 2 | GSTA2 | na | 6075 | 0.181 | -0.0981 | No |
| 3 | GCLM | na | 7095 | 0.150 | -0.0983 | No |
| 4 | GSTA5 | na | 10238 | 0.082 | -0.1729 | No |
| 5 | GSTM5 | na | 10401 | 0.080 | -0.1621 | No |
| 6 | GGT6 | na | 11366 | 0.065 | -0.1773 | No |
| 7 | SMS | na | 11913 | 0.057 | -0.1820 | No |
| 8 | GGCT | na | 12522 | 0.048 | -0.1901 | No |
| 9 | GSTT1 | na | 14076 | 0.030 | -0.2291 | No |
| 10 | GGT7 | na | 14223 | 0.028 | -0.2278 | No |
| 11 | GSTZ1 | na | 15858 | 0.010 | -0.2729 | No |
| 12 | GGT1 | na | 16634 | 0.002 | -0.2948 | No |
| 13 | IDH2 | na | 16744 | 0.001 | -0.2977 | No |
| 14 | GSTO1 | na | 16930 | -0.001 | -0.3029 | No |
| 15 | GSTK1 | na | 17626 | -0.007 | -0.3215 | No |
| 16 | MGST3 | na | 17888 | -0.010 | -0.3271 | No |
| 17 | GSTP1 | na | 18119 | -0.012 | -0.3315 | No |
| 18 | MGST2 | na | 18715 | -0.018 | -0.3452 | No |
| 19 | GPX4 | na | 18963 | -0.020 | -0.3485 | No |
| 20 | GSS | na | 19908 | -0.029 | -0.3701 | No |
| 21 | GPX1 | na | 20965 | -0.040 | -0.3929 | No |
| 22 | GSTO2 | na | 21002 | -0.040 | -0.3861 | No |
| 23 | G6PD | na | 21848 | -0.049 | -0.4010 | No |
| 24 | TXNDC12 | na | 22233 | -0.053 | -0.4017 | Yes |
| 25 | GPX5 | na | 22529 | -0.056 | -0.3992 | Yes |
| 26 | GSTM3 | na | 22649 | -0.058 | -0.3914 | Yes |
| 27 | GPX2 | na | 22846 | -0.060 | -0.3854 | Yes |
| 28 | RRM1 | na | 23408 | -0.067 | -0.3886 | Yes |
| 29 | GSR | na | 24289 | -0.079 | -0.3985 | Yes |
| 30 | GCLC | na | 24925 | -0.089 | -0.3995 | Yes |
| 31 | ODC1 | na | 24985 | -0.090 | -0.3837 | Yes |
| 32 | GSTA3 | na | 25136 | -0.093 | -0.3700 | Yes |
| 33 | PGD | na | 25517 | -0.099 | -0.3618 | Yes |
| 34 | GSTT2 | na | 25629 | -0.100 | -0.3454 | Yes |
| 35 | GSTM1 | na | 25692 | -0.101 | -0.3275 | Yes |
| 36 | GSTA4 | na | 25928 | -0.106 | -0.3137 | Yes |
| 37 | LAP3 | na | 26344 | -0.113 | -0.3037 | Yes |
| 38 | ANPEP | na | 26864 | -0.123 | -0.2947 | Yes |
| 39 | SRM | na | 28051 | -0.150 | -0.2998 | Yes |
| 40 | GPX3 | na | 28069 | -0.150 | -0.2710 | Yes |
| 41 | OPLAH | na | 28271 | -0.155 | -0.2466 | Yes |
| 42 | GSTM2 | na | 28859 | -0.172 | -0.2300 | Yes |
| 43 | IDH1 | na | 29368 | -0.187 | -0.2083 | Yes |
| 44 | GGT5 | na | 29647 | -0.195 | -0.1783 | Yes |
| 45 | GSTM4 | na | 29778 | -0.199 | -0.1433 | Yes |
| 46 | GPX7 | na | 30705 | -0.230 | -0.1252 | Yes |
| 47 | RRM2B | na | 31129 | -0.247 | -0.0894 | Yes |
| 48 | RRM2 | na | 33544 | -0.411 | -0.0791 | Yes |
| 49 | MGST1 | na | 34361 | -0.582 | 0.0106 | Yes |
Table: GSEA details [plain text format]

  

Fig 2: KEGG\_GLUTATHIONE\_METABOLISM      
 Blue-Pink O' Gram in the Space of the Analyzed GeneSet

  

Fig 3: KEGG\_GLUTATHIONE\_METABOLISM: Random ES distribution      
 Gene set null distribution of ES for **KEGG\_GLUTATHIONE\_METABOLISM**
